# Sedentary behaviour (especially accumulation pattern) has an independent negative impact on skeletal muscle size and architecture in community-dwelling older adults

**DOI:** 10.1371/journal.pone.0294555

**Published:** 2024-02-23

**Authors:** Jorgen A. Wullems, Hans Degens, Sabine M. P. Verschueren, Christopher I. Morse, Dale M. Grant, Gladys L. Onambélé-Pearson

**Affiliations:** 1 Department of Sport and Exercise Sciences, Faculty of Science and Engineering, Manchester Metropolitan University, Manchester, United Kingdom; 2 Musculoskeletal Rehabilitation Research Group, Department of Rehabilitation Sciences, KU Leuven, Leuven, Belgium; 3 Department of Life Sciences, Manchester Metropolitan University, Manchester, United Kingdom; 4 Lithuanian Sports University, Kaunas, Lithuania; The University of British Columbia, CANADA

## Abstract

Prolonged sedentary behaviour (SB) i.e. longer bouts, is suggested to have a range of negative health effects, independent of habitual light and medium-to-vigorous physical activity (LIPA or MVPA). Any effect on musculoskeletal size, architecture or morphology has seldom been reported in older adults. Moreover, no study has yet determined if any association would persist following adjustment for covariates. Therefore, the aim of the present study was to investigate the associations between SB, and properties of the *Gastrocnemius Medialis* (GM) muscle, in a cross-sectional sample of older adults using compositional data analysis. 105 healthy older adults (73±6y) wore a thigh mounted tri-axial accelerometer for seven consecutive days, and underwent ultrasound [e.g. muscle length (L_m_), anatomical cross-sectional area (ACSA), muscle volume (V_M_), fascicle length (L_F_), & physiological cross-sectional area (PCSA)], body composition (e.g. DEXA) and health (e.g. medical history) assessments. In-unadjusted models, SB time was negatively associated with ACSA at 75% of L_m_ (R^2^_adj_ = 0.085), V_M_ (R^2^_adj_ = 0.020), and PCSA (R^2^_adj_ = 0.039). Standing was positively associated with pennation angle (R^2^_adj_ = 0.110), which persisted following co-variate adjustment (R^2^_adj_ = 0.296). In fully adjusted models, both SB & LIPA time were associated with ACSA at 75% of L_m_ (Both R^2^_adj_ = 0.393). Standing and light activity time were also associated with L_F_, V_M_, & PCSA (R^2^_adj_ 0.116–0.573). In fully adjusted models, SB pattern parameters (i.e. the manner in which sedentary behaviour is accumulated daily throughout waking hours such as the timing, duration and frequency of sedentary bouts), were associated with *GM* muscle properties (R^2^_adj_ 0.156–0.564) including L_M_, L_F_, and V_M_. The pattern, rather than accumulated daily SB time, was associated with the size and architecture of the *GM*. Our results suggest that regardless of co-existing habitual physical activities, SB bouts should be kept short and frequently interrupted to offset some of the deleterious ageing-related muscle architecture characteristics changes.

## Introduction

Skeletal muscle ageing is a phenomenon characterised by a decrease in muscle mass [1] and strength [2–5], a decrease in the ability to fully activate the muscle [6] and an increase in agonist-antagonist co-contraction [7]. Generally, this results in a decreased functional capacity [8], and increased disability and physical dependence in older people [9–11]. In addition, falls in old age and the resulting fractures have been associated with lower muscle strength and those fractures lead to increased morbidity, higher rate of hospitalisation and mortality [12, 13].

Other than muscle mass and fibre type composition, the power output, force generating capacity and maximal shortening velocity are also influenced by the architecture of the muscle [14–17]. Muscle architecture is often described in terms of fascicle length (L_F_), pennation angle (θ) and physiological cross-sectional area (PCSA), where the latter provides a more accurate measure of the contractile area than muscle anatomical cross-sectional area, especially for pennate muscles [18]. With ageing, not only muscle volume (V_M_), but also fascicle length (L_F_), pennation angle (θ) and muscle physiological cross-sectional area (PCSA) are reduced [19], where the decline in θ aligns muscle fascicles more with the ‘line of pull’ and hence attenuates some of the loss of power and force in old age [17]. Apart from ageing, other factors also have a significant impact on skeletal muscle, such as sex, body composition, and training status, where being a man, being trained or having a higher body mass, are typically associated with larger muscle strength and mass [20–24]. It is also possible that sleep (through its impact on muscle catabolic hormones for instance [25]) could positively affect muscle ageing [26, 27] and as such should be considered in accounting for habitual physical behaviour effects. Thus, it is important to consider some or all these factors when examining any effects on muscle size and architecture in a cross-sectional study of an aged population.

Diminished strength during ageing is especially detrimental in lower body antigravity muscle groups, given their functional relevance for satisfactory performance of activities of daily living (walking, sit-to-stand transitions, stair climbing etc [28]). In-fact, diminished *Triceps Surae* strength [e.g. Gastrocnemius Medialis (GM)] is associated with impaired stability during ambulation [29], reduced walking speed [30], and increased postural sway [2, 29] in older adults. In-fact, improved gait speed following a light activity intervention in older adults, is partially dependant on an increase in GM morphology [Fascicle length (L_F_)] [31].

An ageing-related drop in habitual physical activity (PA) levels is thought to, at least partially, explain the muscle ageing effects [6, 32]. Although evidence is limited, sedentary behaviour (SB) is also suggested to be independently associated with muscle health (herein defined as optimal muscle size, architecture and/or function) [31, 33] Accordingly, Gianoudis *et al*. found, i) TV viewing time (self-reported) is inversely related to total body and leg lean mass, and ii) each 1-hour increase in self-reported SB time is associated with a 33% increased risk of sarcopenia [low appendicular lean mass (ALM) & strength/ function] [34]. A more prolonged SB pattern (i.e. longer SB bout length) also appears important, as the risk of pre-sarcopenia (ALM divided by BMI) decreases by 45%, for every additional 10 sit-to-stand transitions performed per day [35]. Interestingly, this association was attenuated when controlling for co-variates including daily stepping time [35]. SB has also been identified as mediating the association between obesity and falls in older people [36]. Some work even suggests that sarcopenia is catalysed by the amount of visceral and intramuscular fat [21], itself increased through SB [34]. Accordingly, two studies found a positive relationship between objectively determined SB, and greater adiposity (lower limb) in older adults [35, 37]. Counter-intuitively, greater SB was also associated with enhanced ‘muscle quality’ in older adults, which the authors speculated could be due to a loading effect of extra mass caused by adiposity [37]. However, previous studies have only assessed muscle mass using DEXA, which fails to account for key factors like muscle architecture. Furthermore, such studies have only examined the association between SB time and muscle properties in isolation, or with only partial adjustment for time spent in other behaviours (e.g. sleep, light intensity physical activity/LIPA and medium-to-vigorous physical activity/MVPA) [38, 39]. Similarly, only few studies have quantified SB pattern [37, 40, 41], i.e. the manner in which sedentary behaviour is accumulated daily throughout waking hours such as the timing, duration and frequency of sedentary bouts [42], let alone considered their impact on physiological outcomes.

Overall, the literature has suggested several factors that contribute to muscle ageing, in which SB may potentially play a role. To our knowledge, no study has yet comprehensively investigated to what extent SB measurably and negatively affects the ageing-related changes in muscle size and architecture. There is also need for continued investigation of the *Triceps Surae* muscle group [e.g. *Gastrocnemius Medialis* (*GM*)], since properties of this muscle group are associated with functional disability in older adults [2, 29, 43]. Therefore, the aim of the present study was to assess the association between SB, and gastrocnemius muscle size, architecture and morphology in older adults, after accounting for covariates such as age, sex, body composition, habitual physical activity, training status, diet and health parameters. Different objective measures of SB (obtained through accelerometry) were studied, respectively (i) total daily SB and (ii) daily SB patterns. It was hypothesised that in older adults, the continuum of muscle size, architecture and morphology would be linearly associated with total daily SB accumulation, and/or a pattern of SB accumulation such as the length of sedentarism bouts. It was also hypothesised that this relationship would be present regardless of concurrent PA.

## Materials and methods

One hundred and five community-dwelling, healthy older adults (Mean±SD, 72.8±6.0 years old, 166±9 cm body height, 73.0±13.4 body mass, BMI of 25.9±6.0 kg∙m^-2^, 56 women, 104 Caucasian) recruited between 2015 and 2017 participated in this cross-sectional study. Sample size arose from the planned regressions analyses, whereby it has been suggested that there should be at least 25 samples for reliable outcomes [44]. They were recruited both from an existing university database of former studies participants [45, 46], and from the local area via social meetings, posters, and word-of-mouth. This study was approved by the ethical review board of Manchester Metropolitan University, Crewe, UK (Approval code: 12.12.14). All participants provided written informed consent prior to study participation.

Participants were excluded if they were: aged <60 years, diabetic, had any health issue affecting their mobility or ability to exert maximum force with the lower limb muscles, had any recent (<3 months) injury or surgery on their tested leg, not able to understand or follow study instructions, or not competent to make an informed decision about study participation.

Participants attended the university twice. During the first visit they were familiarised with experimental procedures and received an accelerometer to monitor their habitual daily physical behaviour during a week. The second visit included several tests such as a whole-body DEXA scan to measure body composition, health, and lifestyle questionnaires, as well as muscle size and architecture of the gastrocnemius medialis (GM) was assessed.

### SB and PA outcomes: Accelerometer data

SB and PA levels were monitored for seven consecutive days using a triaxial accelerometer. The waterproof accelerometer (43 x 40 x 13 mm, 16 grams, GENEActiv Original, ActivInsights Limited, Kimbolton, Cambridgeshire, UK) was mounted on the anterior mid-thigh of the dominant leg (preferred for single-leg balance) at 50% femur length using transparent film dressing (Tegaderm™, 3M Health Care, St. Paul, MN, USA). The accelerometer was initialised to sample at 60 Hz [47]. Participants were provided with a log sheet and instructed to record their sleeping times, which allowed accurate analysis of awake SB and PA. The accelerometer data was analysed with an in-house developed machine-learning algorithm and software application [48]. This application provides a wide range of daily SB and PA outcomes, such as total time spent in different intensities, time spent in moderate-to-vigorous PA (MVPA) bouts of ≥10 continuous minutes, breaks in SB and distribution of SB bouts (Table 1). These outcomes were adopted from previous studies [40, 49], which provide details on the calculations performed. The accelerometer data was only considered valid, if ≥5 days (of which ≥1 weekend day) were measured [33]. This was the case in 105 out of 106 participants tested. Average values of all outcomes over the valid days were considered for further analyses.

**Table 1:** 3D-Accelerometer-derived physical behaviour outcomes [37, 40, 41]. SB, sedentary behaviour; PA, physical activity; LIPA, light-intensity physical activity; MVPA, moderate-to-vigorous physical activity; ^¶^Daily measure, unless stated otherwise. SB pattern outcomes are specified with *.

Accelerometer outcome^¶^	Description	Mean (SD) or ^¶^median (IQR)	
Sleep (hrs)	Time spent sleeping	8.4 (0.8)^¶^	
SB (hrs)	Time spent in SB	9.3 (1.5)	
Standing (hrs)	Time spent standing	0.7 (0.3)	
LIPA (hrs)	Time spent in LIPA	2.9 (1.0)	
MVPA (hrs)	Time spent in MVPA	2.7 (1.0)	
SB level (low/high)	Daily SB <8 or ≥8 hours	19	86
Breaks SB (n)*	SB interruptions with ≥2 consecutive minutes upright activity	22.2 (3.5)	
Short SB bouts (n)*	SB bouts <30 minutes duration	17.0 (3.8)	
Long SB bouts (n)*	SB bouts ≥30 minutes duration	6.0 (1.2)	
α *	Scaling parameter sedentary bout length distribution	1.45 (0.04)	
X_1/2_ (mins)*	Median SB bout duration	8.8 (11.8)^¶^	
W_1/2_ (%)*	Fraction total sedentary time accumulated in bouts longer than median sedentary bout length	93.3 (11.2)^¶^	
W_50%_ (mins)*	Half of total SB is accumulated in SB bouts ≤ this stated duration	58.3 (22.9)^¶^	
F (bouts∙hrs^-1^)*	Fragmentation index of SB bouts and total SB	2.5 (0.7)^¶^	
Period (mins)*	Mean period between SB bouts	10.2 (2.9)^¶^	
PA bouts (n)	Bouts of ≥2 consecutive minutes upright activity	22.2 (3.5)	
Total PA bouts time (mins)	Total PA bouts duration	365.2 (95.9)	
SB during PA bout (%)	Percent of time spent in SB during PA bouts	1.5 (0.7)^¶^	
Standing during PA bout (%)	Percent of time spent in standing during PA bouts	11.8 (4.6)	
LIPA during PA bout (%)	Percent of time spent in LIPA during PA bouts	44.2 (11.0)	
MVPA during PA bout (%)	Percent of time spent in MVPA during PA bouts	42.5 (12.4)	
MVPA_≥10 mins_ (mins)	Total time spent in ≥10 consecutive minutes MVPA	3.4 (10.6)^¶^	
sMVPA (mins)	Sporadic MVPA (total MVPA—MVPA_≥10 mins_)	153.5 (57.8)	
Physically active (no/yes)	Weekly MVPA_≥10 mins_ <150 or ≥150 mins	94	11

With SB being part of a composition of daily activity behaviours, focusing on SB accumulation alone may lead to erroneous conclusions [38]. Therefore, we aimed to employ both total accumulation data analysis and SB pattern parameters to assess whether these would show any associations with muscle size, morphology, and architecture. More specifically, SB and PA outcomes were analysed as follows: daily SB accumulations and pattern parameters, as well as a variety of PA outcomes, such as percent standing, light-intensity PA (LIPA) or MVPA during PA bouts, or daily sporadic MVPA (sMVPA). Table 1 gives a detailed list of the parameters assessed.

### Anthropometry and body composition

Body height was measured barefoot and to the closest 0.1 cm using a wall-mounted stadiometer (Holtain Ltd., Crymych, UK). Body mass was measured wearing the least clothing as possible and to the nearest 0.1 kg using a digital body mass scale (Seca GmbH & Co. KG., Hamburg, Germany).

Dual-energy X-ray absorptiometry (DEXA) (Hologic Discovery: Vertec Scientific Ltd, UK) was used to determine participants’ body composition, post 10 hours overnight fasting, with maximum 250 mL of water ingestion and post bladder voiding. Scanning was in a supine position using a ~7-minute whole body protocol (effective dose 8.4 μSv), whilst wearing a hospital gown only. Using the built-in scan analysis software (Version 12.4; QDR for Windows, Hologic, Waltham, MA, USA), whole body analysis [50] was performed to determine body compositional outcomes such as percentages of body fat mass, lean body mass and bone mineral content. Appendicular segmental masses were manually identified and assessed to be able to calculate the skeletal muscle index (SMI; appendicular lean mass per squared body height (kg∙m^2^)). Based on percent body fat mass, participants were classified, in terms of adiposity, as either normal or high (<40% or ≥40% in female, while <28% or ≥28% in male) [51].

### Health and lifestyle questionnaires

All participants provided demographics and information about their previous and current PA and medical status via a general questionnaire, Physical activity status was quantified based on the baseline accelerometer data (see Table 1) whereby classification of non-active or active was for weekly continuous MVPA (i.e. ≥10 mins with no interruption) amounting to either <150 or ≥150 mins, respectively. Current Rheumatoid Arthritis diagnosis required ‘Yes/No’ answers. Additionally, information was collected about their smoking status and dietary intake. They also completed a falls risk assessment tool (FRAT), which served as a measure of frailty [52, 53].

### Muscle size

**Fig 1:**
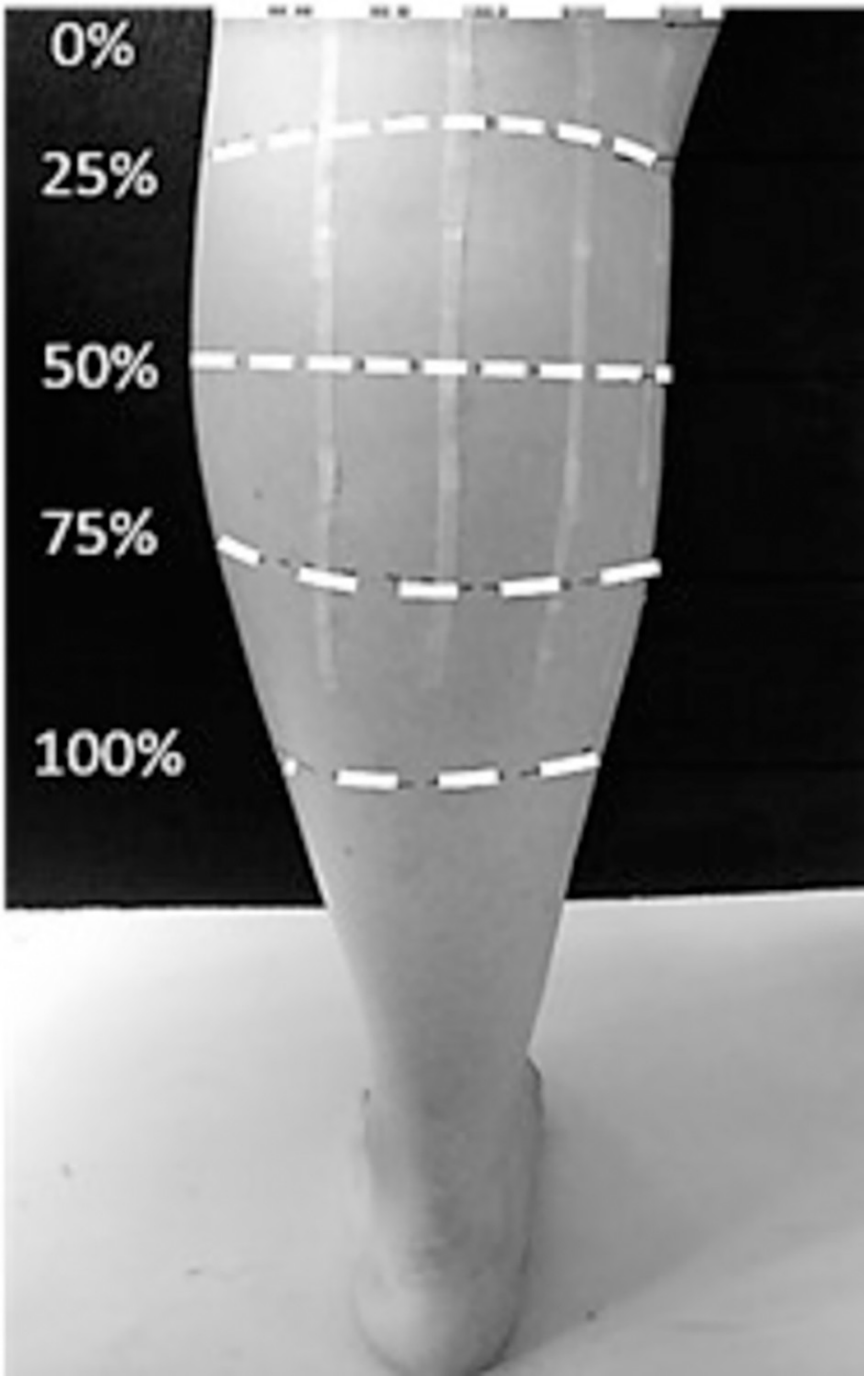
Marking on the lower limb in view of performing ultrasound scanning.

**Fig 2:**
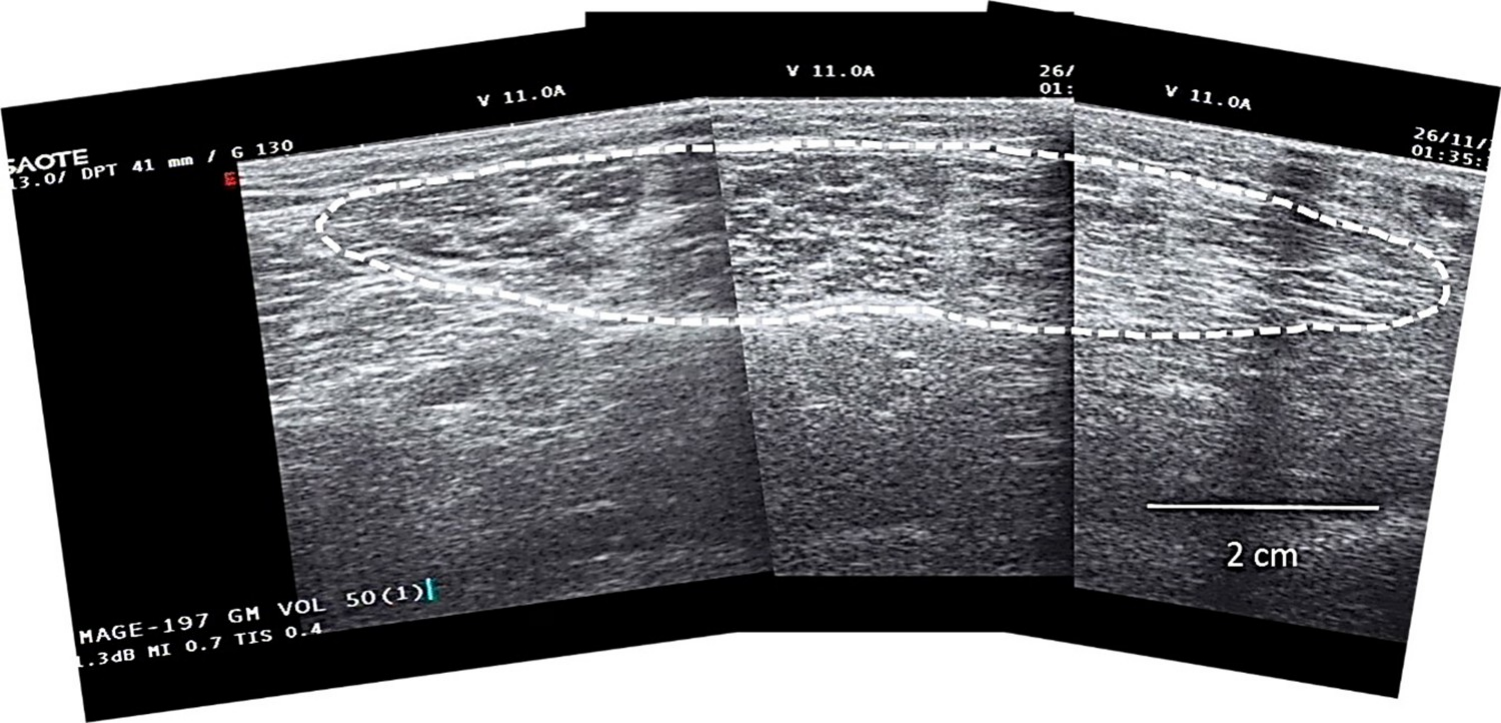
Anatomical cross-sectional area of the gastrocnemius medialis at 50% muscle length.

For the assessment of the size of the gastrocnemius muscle (GM), participants were placed in a prone position with their dominant leg extended and ankle fixed at a 90° angle (the foot at a right angle to the tibia) through strapping using Velcro straps, on a footplate set at this angle and maintained at said angle via both mechanical and electronic ‘stops’. Real-time B-mode ultrasonography (Technos; Esaote S.p.A, Genoa, Italy) was used to assess GM muscle architecture. Firstly, the GM origin (0% GM length) and Achilles tendon insertion into the calcaneus were determined and marked by scanning these sites in the sagittal plane. The distance between these two sites represented the muscle-tendon unit length (L_MTU_; cm). Next, the myotendinous junction was determined as the point of insertion of the GM into the GM tendon through sagittal plane ultrasonography. Muscle length (L_M_; cm) was defined as the distance between GM origin and the myotendinous junction (0–100% GM length). The skin over the GM was then marked at 25, 50 and 75% GM length. Thin strips (~2 mm) of tape (Transpore, 3M, USA) were placed in axillary lines (~3.5 cm apart) along the GM length and across the three marked muscle lengths (Fig 1). They served as echo-absorptive markers for the reconstruction of the anatomical cross-sectional area (ACSA) at each of these muscle sites. Water-soluble transmission gel (Aquasonic 100; Parker Laboratories Inc., Fairfield, NJ, USA) was placed over the ultrasound probe head to improve acoustic coupling during ultrasound scanning. Each section was then transversally scanned across the marked pathway from the medial to lateral GM border, during which the ultrasound probe (7.5-MHz linear-array probe, 3.8 cm wide) was held perpendicular to the skin for the duration of the scanning procedure. While moving the probe steadily, minimal pressure was applied to avoid compression of the muscle tissue. The ultrasound pictures were recorded in real time onto the ultrasound memory then transferred to a computer (at 25 Hz) using capturing software (Adobe Premier Pro version 6), which allowed offline extraction of individual frames. The shadows projected by the micropore tape and anatomical markers were used to reconstruct the ACSAs at each of the three GM lengths of interest (25 (ACSA25), 50 (ACSA50) and 75% (ACSA75)) with photo editing software (Adobe Photoshop Elements, version 10) (Fig 2). The complete ACSAs were measured (cm^2^) using digitising software [54, 55] (ImageJ 1.45; National Institutes of Health, Bethesda, MD, USA). Finally, muscle volume (V_M_; cm^3^) was calculated using the truncated cones method, which required the three measured ACSAs plus two assumed ACSAs at the GM origin (0%) and insertion (100%). For the latter two, a standard area of 0.5 cm^2^ was used [21]. In total, the volumes of four different cones (0–25, 25–50, 50–75, and 75–100%) were calculated and summed for the muscle volume. This approach to assess anatomical cross-sectional areas [21] by stitching several images to reconstruct ACSA [56] and applying the truncated cones to quantify muscle volume [57] to calculate muscle volume [58] has been previously investigated for comparability relative to other approaches. The calculation of each cone volume was therefore carried out using the following formula:

Cone volume (cm^3^) = (*h*/3) * (*ACSA*_*base*_ + √(*ACSA*_*base*_ * *ACSA*_*top*_) + *ACSA*_*top*_)

where *h* = distance between the segments (cm), *ACSA*_*base*_ = anatomical cross-sectional area (cm^2^) of the cone base, and *ACSA*_*top*_ = anatomical cross-sectional area (cm^2^) of the cone top.

### Muscle architecture

**Fig 3:**
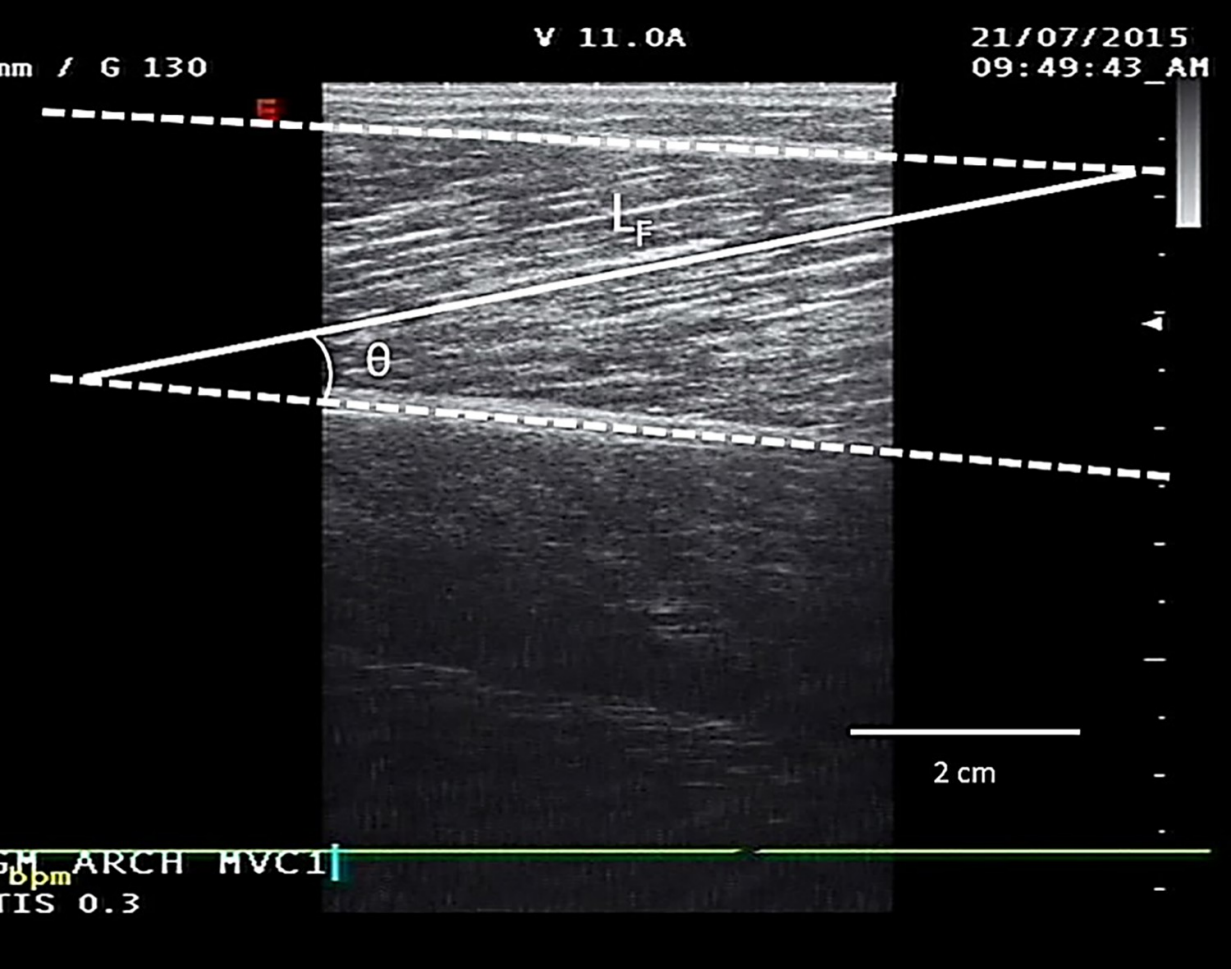
Muscle architecture at 50% gastrocnemius medialis muscle length. LF, fascicle length; θ, pennation angle. Upper dashed line represents superficial aponeurosis, bottom dashed line represents deep aponeurosis.

Architecture of the GM of each participant’s self-perceived dominant leg (preferred for single leg balance) was measured with real-time B-mode ultrasonography while participants were seated in an isokinetic dynamometer chair (Cybex Norm; Cybex International, New York, NY, USA) with their hip at 85° angle, self-perceived dominant leg extended, and foot secured to the footplate of the dynamometer at 90°. Non-extending straps were used at the hip, distal thigh, and chest to prevent extraneous movements. Resting measures of L_F_ and θ were obtained by placing the ultrasound probe perpendicular to the dermal surface in the mid-sagittal plane at 50% of the GM muscle length (Fig 3). Again, water-soluble transmission gel was placed over the ultrasound probe to improve acoustic coupling during ultrasound scanning. The ultrasound picture was recorded in real time onto a computer using capturing software, from where individual images were extracted for post-testing analyses. L_F_ and θ were analysed on these images using digitising software (Image J, as above). To do so, three fascicles had to be clearly visible in the area between the deep and superficial aponeuroses. L_F_ (cm) and θ (°) (defined as the angle between a fascicle’s orientation and the tendon axis) were measured for all three fascicles, with the mean value recorded as the participant’s data. In cases where a chosen fascicle extended beyond the scanning window, linear extrapolation was applied, but only if ≥60% of the fascicle was visible [19]. These extrapolations have previously been shown to be valid [59].

Following the measurement of L_M_, L_F_ and V_M_, calculation of normalised fascicle length (L_F-N_) and resting PCSA (cm^2^) was performed. The first was done by dividing L_F_ (cm) by L_M_ (cm), while for the second V_M_ (cm^3^) was taken over L_F_ (cm). Architecture outcomes are summarised in Table 2.

**Table 2:** Descriptive statistics and resting gastrocnemius medialis muscle size and architecture. GM, gastrocnemius medialis; SD, standard deviation; IQR, interquartile range; L_MTU_, muscle-tendon unit length; L_M_, muscle length; ACSA, anatomical cross-sectional area; V_M_, muscle volume; L_F_, fascicle length; L_F-N_, normalised fascicle length; θ, fascicle pennation angle; PCSA, physiological cross-sectional area.

	Variable	Mean (SD) or ^¶^median (IQR)
**Descriptive Statistics**	Age (yrs.)	72.8 (6.0)
	Sex (female / male)	56 vs 49
	Ethnicity (white / black)	104 vs 1
	Body height (cm)	166.3 (9.3)
	Body mass (kg)	73.0 (13.4)
	BMI (kg∙m^-2^)	25.9 (6.0)^**¶**^
	Body fat mass (%)	36.3 (7.9)
	Body lean mass (%)	60.2 (7.5)
	Body BMC (%)	3.5 (0.7)
	SMI (kg∙m^-2^)	6.4 (1.8)^**¶**^
**Resting GM variables**	L_MTU_ (cm)	40.1 (3.5)
	L_M_ (cm)	22.3 (3.2)
	ACSA25 (cm^2^)	11.6 (4.4)^¶^
	ACSA50 (cm^2^)	15.0 (5.3)^¶^
	ACSA75 (cm^2^)	8.8 (4.6)^¶^
	V_M_ (cm^3^)	186 (83)^¶^
	L_F_ (cm)	7.4 (1.2)
	L_F-N_	0.34 (0.06)
	θ (°)	15.2 (2.5)
	PCSA (cm^2^)	26.0 (10.0)^¶^

### Reliability and minimisation of bias

Test-retest inter-day reliability for ultrasound scanning was investigated in a sub-sample of 20 participants (from the total study population) by intraclass correlation coefficients (ICCs) for absolute agreement using a two-way mixed model, with 95% Confidence Interval. Reliability values <0.5 were interpreted as poor, between 0.5–0.75 as moderate, between 0.75–0.9 as good, and >0.9 as excellent [60]. ICCs were L_M_ = 0.941, ACSA25 = 0.824, ACSA50 = 0.910, ACSA75 = 0.974, L_F_ = 0.700 and θ = 0.645. In our laboratory, the inter-day systematic error (SE) and typical error (TE) values are ACSA25 (SE: 14%, TE: 46.25 cm^2^), ACSA50 (SE: 9%, TE: 42.00 cm^2^), ACSA75 (SE: 12%, TE: 28.01 cm^2^), L_F_ (SE: 9%, TE: 4.9cm), and θ (SE: 6.25%, TE: 1.38°).

All data were recorded using a unique code that maintained the anonymity of each participant. All data were reduced by the same researcher to avoid any inter-rater variability issues.

### Statistical analyses

All statistical analyses were performed using IBM SPSS Statistics for Windows, version 24.0 (IBM Corp., Armonk, NY, USA) and p-values <0.05 were considered statistically significant.

Prior to conducting any inferential statistical analysis, all outcome variables were checked for normality (either Kolmogorov-Smirnov or Shapiro-Wilk test). In case of non-normality, the variables were log-transformed and the distribution of the transformed data also checked. The outcome variables are displayed as mean (standard deviation (SD)) or median (interquartile range (IQR)) depending on parametricity status.

Potential covariates were analysed per outcome variable by running a univariate General Linear Model (GLM) (Table 3, S1 Table).

**Table 3:** Correlation coefficients during covariate analysis. L_MTU_, muscle-tendon unit length; L_M_, muscle length; ACSA, anatomical cross-sectional area; V_M_, muscle volume; L_F_, fascicle length; L_F-N_, normalised fascicle length; θ, fascicle pennation angle; PCSA, physiological cross-sectional area; BMI, body mass index; PA, physical activity; SB, sedentary behaviour; LIPA, light-intensity physical activity; MVPA, moderate-to-vigorous physical activity; sMVPA, sporadic moderate-to-vigorous physical activity; ^¶^Log-transformed. Bold values represent significances at P<0.05 level.

	L_MTU_	L_M_	ACSA25^¶^	ACSA50^¶^	ACSA75^¶^	V_M_^¶^	L_F_	L_F-N_	θ	PCSA^¶^
Age (yrs.)	-0.037	**-0.287**	**-0.230**	**-0.193**	-0.171	**-0.289**	-0.158	0.115	0.032	**-0.258**
Sex	**0.649**	**0.338**	0.167	**0.226**	**0.304**	**0.339**	0.144	-0.161	0.087	**0.338**
Body height (cm)	**0.799**	**0.491**	**0.282**	**0.235**	**0.235**	**0.416**	**0.207**	**-0.220**	-0.035	**0.385**
Body mass (kg)	**0.508**	**0.347**	**0.500**	**0.580**	**0.487**	**0.593**	0.174	-0.142	**0.286**	**0.619**
BMI (kg∙m^-2^)	0.032	0.055	**0.361**	**0.478**	**0.379**	**0.374**	0.035	-0.026	**0.347**	**0.433**
PA bouts (n)	0.129	0.140	0.040	0.112	0.104	0.137	**0.211**	0.072	-0.020	0.053
Total PA bouts time (mins)	-0.083	0.008	0.065	-0.090	-0.048	-0.021	0.170	0.167	-0.095	-0.119
SB during PA bout (%)	-0.184	-0.097	-0.172	-0.127	**-0.200**	-0.180	-0.026	0.068	-0.094	**-0.206**
Standing during PA bout (%)	-0.111	0.049	0.023	0.084	0.100	0.088	-0.041	-0.077	**0.267**	0.125
LIPA during PA bout (%)	**-0.269**	-0.055	0.005	-0.006	-0.022	-0.029	0.007	0.053	-0.053	-0.033
MVPA during PA bout (%)	**0.291**	0.036	-0.004	-0.019	-0.007	0.003	0.011	-0.022	-0.046	-0.006
MVPA_≥10 mins_ (mins)	0.042	0.007	0.108	-0.031	0.067	0.033	-0.004	-0.020	-0.003	0.040
sMVPA (mins)	0.177	0.049	0.039	-0.047	-0.040	0.006	0.123	0.084	-0.088	-0.064
Physical activity status	0.052	-0.006	0.036	-0.032	0.095	0.016	-0.048	-0.037	0.001	0.045

Since daily time spent in sleep, SB and physical activity (PA) is constrained to 24 hours, we used compositional data analysis for these accelerometer outcomes. This type of analysis has been described in detail previously [38, 39]. Briefly, daily compositions are transformed into isometric log-ratio coordinates, which are then unconstrained and allow the application of traditional multivariate statistics. Thus, both single and multiple linear regression analyses were used to study the associations with SB levels, proportional total daily SB and PA, and daily SB pattern parameters. The covariates identified via bivariate correlations (Table 3, S1 Table) were added to the regression models using backward elimination, where a parameter was retained if p <0.20 [38] thereby accounting for the joint impact of all predictors (on the final model) but also ensuring that only important predictors would remain in the model. For all models, Durbin-Watson statistics (>1.0 and <3.0) were checked to identify any correlation between the predictor and covariates, and covariates with Variance Inflation Factor ≥10.0 were removed from the regression model, one at a time. The same was done with individual cases showing Cook’s distance ≥1.0 (the influence of individual cases on the resulting model) thereby minimising the impact of any outlier if present. Ultimately, the amount of explained variance (R^2^) was used as a measure of effect size using Falk & Miller’s (1992) thresholds so that 0.01, 0.09, and 0.25 denoted small, medium, and large effects, respectively.

## Results

### Descriptive statistics

Table 2. shows the study sample’s descriptive statistics for GM size and architecture, in the entire study sample (n = 105).

### Covariate analysis

The variables identified as covariates included: age, sex, body height, body mass, body mass index (BMI), skeletal muscle index (SMI), body fat mass, body lean mass, body bone mineral content (BMC), adiposity class, falls risk assessment tool (FRAT) score, menopause age, history of major illness, current resistance training, intake of dairy products, current rheumatoid arthritis diagnosis, calcium/vitamin D supplement usage, number of daily PA bouts, SB during PA bouts, standing during PA bouts, light-intensity PA (LIPA) during PA bouts and MVPA during PA bouts (Table 3, S1 Table).

### Daily total SB and PA- unadjusted compositional models

Compositional data analysis showed significant associations between time spent in some physical behaviours relative to total awake time, for several muscle size and architecture outcomes (Table 4).

**Table 4:** Regression analysis results for daily total sedentary and physical activity behaviour accumulations. L_MTU_, muscle-tendon unit length; L_M_, muscle length; ACSA, anatomical cross-sectional area; V_M_, muscle volume; L_F_, fascicle length; L_F-N_, normalised fascicle length; θ, fascicle pennation angle; PCSA, physiological cross-sectional area; SB, sedentary behaviour; LIPA, light-intensity physical activity; MVPA, moderate-to-vigorous physical activity; ^¶^Log-transformed; *P<0.05; ** P<0.01.

		Without covariates		With covariates	
		B	R^2^_adj_	B	R^2^_adj_
L_MTU_	Sleep	-1.22	0.063*	1.00	0.831**
	SB	1.70		-0.26	
	Standing	-0.95		-0.06	
	LIPA	-1.38		-0.40	
	MVPA	**1.85**		-0.29	
L_M_	Sleep	-1.43	-0.035	1.72	0.585**
	SB	0.97		-1.89	
	Standing	0.17		0.85	
	LIPA	-0.08		-0.34	
	MVPA	0.37		-0.35	
ACSA25^¶^	Sleep	-0.37	-0.009	-0.07	0.318**
	SB	0.22		-0.08	
	Standing	0.03		0.04	
	LIPA	0.07		0.11	
	MVPA	0.05		0.00	
ACSA50^¶^	Sleep	**-0.64**	0.038	-0.35	0.426**
	SB	**0.52**		0.20	
	Standing	0.07		0.07	
	LIPA	0.05		0.16	
	MVPA	0.00		-0.07	
ACSA75^¶^	Sleep	**-0.96**	0.085*	**-0.78**	0.393**
	SB	**0.73**		**0.41**	
	Standing	0.15		0.13	
	LIPA	0.06		**0.28**	
	MVPA	0.02		-0.04	
V_M_^¶^	Sleep	**-0.68**	0.020	-0.17	0.578**
	SB	**0.50**		0.02	
	Standing	0.09		**0.13**	
	LIPA	0.05		0.08	
	MVPA	0.04		-0.06	
L_F_	Sleep	-1.00	-0.001	-1.13	0.116**
	SB	0.22		0.36	
	Standing	-0.09		-0.19	
	LIPA	0.51		**0.83**	
	MVPA	0.37		0.11	
L_F-N_	Sleep	-0.04	-0.003	-0.07	0.224**
	SB	0.00		0.04	
	Standing	-0.01		-0.01	
	LIPA	0.03		**0.04**	
	MVPA	0.01		0.01	
θ	Sleep	**-5.16**	0.110**	-3.22	0.296**
	SB	**4.24**		2.63	
	Standing	**1.84**		**1.70**	
	LIPA	-0.67		-0.32	
	MVPA	-0.25		-0.80	
PCSA^¶^	Sleep	**-0.53**	0.039	-0.20	0.573**
	SB	**0.46**		0.15	
	Standing	0.10		**0.13**	
	LIPA	-0.01		0.02	
	MVPA	-0.02		-0.10	

MVPA was associated with L_MTU_ (β = 0.21, R^2^_adj_ = 0.063). Similarly, both sleep (β = -0.47) and SB (β = 0.60) (both R^2^_adj_ = 0.038) were associated with ACSA50. In parallel, sleep (β = -0.60) and SB (β = 0.71) (both R^2^_adj_ = 0.085) were also associated with ACSA75. Markedly also, both sleep (β = -0.43) and SB (β = 0.50) (both R^2^_adj_ = 0.020) were associated with V_M_.

In terms of GM muscle architecture, sleep (β = -0.45) was negatively, whilst SB (β = 0.58) and standing (β = 0.33) (all R^2^_adj_ = 0.110) were positively associated with θ. Similarly, sleep was negatively (β = -0.41) and SB was positively (β = 0.55) (both R^2^_adj_ = 0.039) associated with PCSA.

### Daily total SB and PA- covariate adjusted compositional models

When adjusting the regression models for the identified covariates, the above associations changed significantly. Whilst ACSA75 as in the previous model, still showed associations with sleep (negatively; β = -0.49) and SB (positively; β = 0.41), now also LIPA (β = 0.27) was positively associated with this variable (all R^2^_adj_ = 0.393). Here, only standing (β = 0.17, R^2^_adj_ = 0.578) was associated with V_M_.

In terms of muscle architecture parameters, LIPA exhibited associations with L_F_ (β = 0.24, R^2^_adj_ = 0.116) and L_F-N_ (β = 0.21, R^2^_adj_ = 0.224). Similarly, standing exhibited associations with both θ (β = 0.31, R^2^_adj_ = 0.296) and PCSA (β = 0.21, R^2^_adj_ = 0.573).

### Daily SB pattern parameters- unadjusted compositional models

Significant associations with muscle architecture outcomes were found for SB pattern parameters (Table 5). Thus, L_M_ was negatively associated with median SB bout duration (X_1/2_) (β = -0.20, R^2^_adj_ = 0.030), while L_F_ was positively associated with Breaks in SB, Short SB bouts, and bout frequency (F) (β = 0.21, R^2^_adj_ = 0.035; β = 0.21, R^2^_adj_ = 0.036; and β = 0.22, R^2^_adj_ = 0.041 respectively), but negatively associated with ‘Half of total SB accumulated in SB bouts ≤ a stated duration’ (W_50%_) (β = -0.24, R^2^_adj_ = 0.047).

**Table 5:** Regression analysis results for daily sedentary behaviour pattern parameters. L_MTU_, muscle-tendon unit length; L_M_, muscle length; ACSA, anatomical cross-sectional area; V_M_, muscle volume; L_F_, fascicle length; L_F-N_, normalised fascicle length; θ, fascicle pennation angle; PCSA, physiological cross-sectional area; Breaks SB, sedentary behaviour interruptions with ≥2 consecutive minutes upright activity; Short SB bouts, sedentary behaviour bouts <30 minutes duration; Long SB bouts, sedentary behaviour bouts ≥30 minutes duration; α, scaling parameter sedentary bout length distribution; X_1/2_, median SB bout duration; W_1/2_, fraction total sedentary time accumulated in bouts longer than median sedentary bout length; W_50%_, half of total SB is accumulated in SB bouts ≤ this duration; F, fragmentation index of SB bouts and total SB; Period, mean period between SB bouts; 95%-CI, 95% confidence interval; ^¶^Log-transformed; *P<0.05; ** P<0.01.

		Without covariates				With covariates			
		B	95%-CI		R^2^_adj_	B	95%-CI		R^2^_adj_
L_MTU_	Breaks SB	0.13	-0.07	0.33	0.007	0.06	-0.02	0.15	0.835**
	Short SB bouts	0.06	-0.12	0.24	-0.006	0.05	-0.02	0.13	0.833**
	Long SB bouts	0.51	-0.04	1.05	0.023	0.05	-0.20	0.30	0.833**
	α	-5.52	-22.15	11.12	-0.005	0.51	-6.62	7.65	0.833**
	X_1/2_	-0.01	-0.02	0.00	0.017	0.00	-0.01	0.00	0.834**
	W_1/2_	0.03	-0.07	0.12	-0.007	0.02	-0.02	0.06	0.833**
	W_50%_	-0.02	-0.06	0.01	0.004	-0.01	-0.02	0.01	0.832**
	F	-0.18	-1.27	0.91	-0.009	0.27	-0.19	0.74	0.832**
	Period	-0.20	-0.48	0.08	0.010	-0.10	-0.22	0.02	0.837**
L_M_	Breaks SB	0.13	-0.05	0.31	0.010	**0.13**	**0.01**	**0.25**	0.590**
	Short SB bouts	0.10	-0.07	0.26	0.004	0.11	0.00	0.22	0.587**
	Long SB bouts	0.14	-0.36	0.64	-0.007	0.01	-0.36	0.39	0.576**
	α	2.94	-12.24	18.12	-0.008	8.05	-2.33	18.43	0.584**
	X_1/2_	**-0.01**	**-0.02**	**0.00**	0.030*	-0.00	-0.01	0.00	0.580**
	W_1/2_	-0.01	-0.10	0.08	-0.009	-0.03	-0.09	0.04	0.579**
	W_50%_	-0.03	-0.06	0.00	0.019	**-0.04**	**-0.06**	**-0.01**	0.607**
	F	0.23	-0.76	1.22	-0.008	**0.84**	**0.16**	**1.53**	0.594**
	Period	-0.03	-0.29	0.23	-0.009	-0.00	-0.19	0.19	0.576**
ACSA25^¶^	Breaks SB	0.00	-0.01	0.02	-0.008	0.00	-0.01	0.01	0.298**
	Short SB bouts	0.00	-0.01	0.01	-0.009	0.00	-0.01	0.01	0.300**
	Long SB bouts	0.01	-0.03	0.05	-0.009	-0.02	-0.06	0.01	0.309**
	α	-0.13	-1.34	1.09	-0.009	-0.24	-1.27	0.80	0.293**
	X_1/2_	0.00	0.00	0.00	-0.008	0.00	0.00	0.00	0.302**
	W_1/2_	0.00	-0.01	0.00	-0.002	0.00	-0.01	0.01	0.297**
	W_50%_	0.00	0.00	0.00	-0.006	0.00	0.00	0.00	0.304**
	F	0.00	-0.08	0.08	-0.010	0.05	-0.02	0.12	0.311**
	Period	0.00	-0.02	0.02	-0.009	0.01	0.00	0.03	0.314**
ACSA50^¶^	Breaks SB	0.01	-0.01	0.03	0.003	0.01	0.00	0.02	0.425**
	Short SB bouts	0.00	-0.01	0.02	-0.007	0.01	0.00	0.02	0.422**
	Long SB bouts	0.04	-0.01	0.08	0.016	0.00	-0.04	0.04	0.408**
	α	0.16	-1.26	1.57	-0.009	0.57	-0.58	1.72	0.414**
	X_1/2_	0.00	0.00	0.00	-0.007	0.00	0.00	0.00	0.406**
	W_1/2_	-0.01	-0.01	0.00	0.010	0.00	-0.01	0.00	0.411**
	W_50%_	0.00	0.00	0.00	-0.010	0.00	0.00	0.00	0.415**
	F	-0.03	-0.12	0.06	-0.006	0.05	-0.02	0.13	0.419**
	Period	-0.02	-0.04	0.01	0.010	0.00	-0.02	0.02	0.408**
ACSA75^¶^	Breaks SB	0.01	-0.01	0.03	0.001	0.01	-0.01	0.02	0.288**
	Short SB bouts	0.00	-0.01	0.02	-0.007	0.01	-0.01	0.02	0.287**
	Long SB bouts	0.04	-0.01	0.10	0.014	0.00	-0.05	0.05	0.282**
	α	0.41	-1.23	2.05	-0.007	0.52	-0.87	1.92	0.286**
	X_1/2_	0.00	0.00	0.00	0.015	0.00	0.00	0.00	0.286**
	W_1/2_	-0.01	-0.01	0.00	0.002	0.00	-0.01	0.01	0.283**
	W_50%_	0.00	0.00	0.00	-0.009	0.00	-0.01	0.00	0.296**
	F	-0.02	-0.13	0.08	-0.008	0.05	-0.04	0.15	0.291**
	Period	-0.01	-0.04	0.01	0.001	0.01	-0.01	0.04	0.289**
V_M_^¶^	Breaks SB	0.01	-0.01	0.03	0.009	0.01	-0.00	0.03	0.564**
	Short SB bouts	0.01	-0.01	0.03	-0.001	0.01	-0.00	0.02	0.563**
	Long SB bouts	0.03	-0.02	0.09	0.005	-0.00	-0.04	0.04	0.549**
	α	0.31	-1.31	1.93	-0.008	0.60	-0.55	1.75	0.554**
	X_1/2_	0.00	0.00	0.00	0.009	-0.00	-0.00	0.00	0.557**
	W_1/2_	-0.01	-0.01	0.00	0.003	-0.00	-0.01	0.00	0.553**
	W_50%_	0.00	-0.01	0.00	0.000	**-0.00**	**-0.01**	**-0.00**	0.564**
	F	0.00	-0.11	0.10	-0.010	0.08	-0.00	0.15	0.565**
	Period	-0.01	-0.04	0.02	-0.003	0.00	-0.02	0.02	0.549**
L_F_	Breaks SB	**0.07**	**0.01**	**0.14**	0.035*	**0.09**	**0.02**	**0.15**	0.164**
	Short SB bouts	**0.07**	**0.01**	**0.12**	0.036*	**0.08**	**0.02**	**0.13**	0.159**
	Long SB bouts	-0.06	-0.25	0.12	-0.005	0.00	-0.18	0.18	0.155**
	α	5.31	-0.17	10.79	0.025	3.94	-1.51	9.40	0.167**
	X_1/2_	0.00	-0.01	0.00	-0.004	0.00	0.00	0.01	0.174**
	W_1/2_	0.00	-0.04	0.03	-0.009	**-0.03**	**-0.07**	**0.00**	0.186**
	W_50%_	**-0.01**	**-0.03**	**0.00**	0.047*	-0.01	-0.02	0.00	0.172**
	F	**0.42**	**0.06**	**0.77**	0.041*	**0.44**	**0.10**	**0.78**	0.156**
	Period	0.03	-0.07	0.12	-0.006	0.04	-0.06	0.13	0.160**
L_F-N_	Breaks SB	0.00	0.00	0.00	-0.004	0.00	0.00	0.00	0.214**
	Short SB bouts	0.00	0.00	0.00	-0.001	0.00	0.00	0.00	0.213**
	Long SB bouts	0.00	-0.01	0.00	0.003	0.00	-0.01	0.01	0.208**
	α	0.18	-0.09	0.44	0.007	0.11	-0.12	0.35	0.215**
	X_1/2_	0.00	0.00	0.00	-0.010	0.00	0.00	0.00	0.218**
	W_1/2_	0.00	0.00	0.00	-0.009	0.00	0.00	0.00	0.209**
	W_50%_	0.00	0.00	0.00	-0.005	0.00	0.00	0.00	0.213**
	F	0.02	0.00	0.03	0.020	0.01	-0.01	0.02	0.217**
	Period	0.00	0.00	0.01	-0.003	0.00	0.00	0.00	0.209**
θ	Breaks SB	-0.01	-0.15	0.12	-0.009	0.01	-0.11	0.14	0.278**
	Short SB bouts	-0.04	-0.17	0.08	-0.005	0.00	-0.11	0.11	0.278**
	Long SB bouts	0.27	-0.11	0.66	0.010	0.13	-0.23	0.48	0.281**
	α	-8.87	-20.51	2.76	0.012	-7.42	-17.41	2.58	0.293**
	X_1/2_	0.00	-0.01	0.01	-0.005	0.00	0.00	0.00	0.280**
	W_1/2_	-0.03	-0.10	0.04	-0.004	-0.01	-0.07	0.05	0.278**
	W_50%_	0.02	-0.01	0.04	0.008	0.02	0.00	0.04	0.286**
	F	-0.55	-1.31	0.20	0.010	-0.23	-0.93	0.47	0.281**
	Period	-0.13	-0.33	0.06	0.008	-0.08	-0.26	0.10	0.284**
PCSA^¶^	Breaks SB	0.00	-0.01	0.02	-0.007	0.00	-0.01	0.02	0.536**
	Short SB bouts	0.00	-0.01	0.01	-0.010	0.00	-0.01	0.01	0.536**
	Long SB bouts	0.04	0.00	0.08	0.020	0.00	-0.03	0.03	0.534**
	α	-0.35	-1.69	1.00	-0.007	-0.03	-1.01	0.95	0.534**
	X_1/2_	0.00	0.00	0.00	0.028	0.00	0.00	0.00	0.538**
	W_1/2_	-0.01	-0.01	0.00	0.007	0.00	-0.01	0.00	0.535**
	W_50%_	0.00	0.00	0.00	-0.010	0.00	0.00	0.00	0.534**
	F	-0.06	-0.14	0.03	0.006	0.01	-0.05	0.08	0.534**
	Period	-0.02	-0.04	0.01	0.008	0.00	-0.02	0.02	0.534**

### Daily SB pattern parameters- covariate adjusted compositional models

As seen above, adding covariates to the regression models changed identified associations significantly. Thus, more breaks in SB (β = 0.14, R^2^_adj_ = 0.590) and higher F (β = 0.17, R^2^_adj_ = 0.594), were positively associated with L_M_. Similarly, more Breaks in SB (β = 0.25, R^2^_adj_ = 0.164), Short SB bouts (β = 0.24, R^2^_adj_ = 0.159), and higher F (β = 0.24, R^2^_adj_ = 0.156), were positively associated with L_F_. On the other hand, higher W_50%_ was negatively associated with L_M_ (β = -0.21, R^2^_adj_ = 0.607). as well as VM (β = -0.16, R^2^_adj_ = 0.564). Last but not least, higher W_1/2_ (β = -0.20, R^2^_adj_ = 0.186) was negatively associated with L_F_.

It is again notable that the adjusted R^2^ values for the latter regression models including covariates, varied from 0.155 through 0.607. The effect sizes for the other regression models with covariates, were 0.208 ≤ R^2^_adj_ ≤ 0.837, therefore indicating large effect sizes throughout.

## Discussion

We have identified associations between measures of SB (amount and pattern) and skeletal muscle size, morphology and architecture in older adults both with non-adjusted regression models, and after correcting for covariates. We hypothesised that in older adults, the continuum of muscle size, architecture and morphology would be linearly associated with total daily SB accumulation, and/or a pattern of SB accumulation such as the length of sedentarism bouts. It was also hypothesised that this relationship would be present regardless of concurrent PA. Total daily SB was not associated with GM muscle size and architecture in this group of older people. Surprisingly, total daily SB time relative to other daily behaviours, showed a positive (instead of the hypothesised negative) association with ACSA at 75% GM length. However, in agreement with our hypothesis, L_M_ was positively associated with more breaks in SB, bouts of longer duration that make up 50% of total daily SB and higher ratio of SB bouts to total SB. In addition, V_M_ was negatively associated with SB bouts of longer duration that make up 50% of total daily SB. As for L_F_, it increased with a higher number of either SB breaks, SB bouts <30 minutes duration or ratio of SB bouts to total SB, and it decreased with a greater fraction of total sedentary time accumulated in bouts longer than the median sedentary bout length. As such, the first hypothesis was partially upheld. It was also hypothesised that these effects would occur regardless of concurrent PA. In fully adjusted models (including PA), SB was again unexpectedly positively associated with ACSA at 75% of L_m_ (Both R^2^_adj_ = 0.393). Furthermore, a number of SB pattern parameters, were associated with numerous *GM* muscle properties (R^2^_adj_ 0.156–0.564) including L_M_, L_F_, and V_M_, after accounting for co-variates. Therefore, the second hypothesis was also partially upheld for select *GM* muscle properties. Overall, these findings show that long bouts of SB with little interruptions are negatively associated with some aspects of muscle architecture, which can be counteracted by performing regular light physical activity.

Aside from SB, our data also highlight that more proportional time spent sleeping is associated with a decreased ACSA at 75% GM length in older people, while the opposite occurs with time spent in LIPA relative to other daily behaviours.

Notably, the observation of increased ACSA75 with more time spent in SB relative to other behaviours seems counter-intuitive. Tomlinson et al. [21] found positive correlations in the older people (all *r* ≥0.39) between measures of body composition (e.g. BMI, body mass and fat mass) and GM θ, V_M_ and PCSA. It was thus understood that increased fat mass could induce extra loading on skeletal muscles of the lower limb [61], resulting in higher absolute muscle strength, possibly due to greater total body mass [62]. A larger body mass however is unlikely to explain our current results (increased ACSA75 with more time spent in SB relative to other behaviours) given that we accounted for covariates in the current regression models. Since both SB and ageing potentially result in skeletal muscle fat infiltration, this could also explain an increase in ACSA75. In the current investigation, muscle morphology was determined, but not composition (i.e. intrinsic ratio of skeletal muscle, to fat, to collagenous tissue). Thus, it is possible that the increase in ACSA is due to fat infiltration rather than muscle tissue growth [63] (i.e. a pseudo-hypertrophy) with previously observed negative impact on muscle function in the older people. Certainly, this needs future MRI-based studies, as it seems reasonable to anticipate a poor validity of ultrasound volume measures in the older person especially, as they are likely to exhibit intramuscular fat infiltration. Interestingly, most of the identified associations with compositional data analysis (75.0%) incorporate either standing or LIPA suggesting a potential marked linear impact of such physical behaviour in this age group.

Apart from total accumulated daily SB, it is also important to focus on daily SB patterns, as total amounts could be similar but patterns of accumulations different. Generally, our results did show a number of effects of SB pattern parameters on GM muscle size, morphology and architecture, namely for L_M_, V_M_ and L_F_. Lm appeared to increase with better daily SB patterns, in this case more SB breaks, shorter SB bout durations making up 50% of total SB and higher ratio of SB bouts to total SB. Similarly, V_M_ was found to increase when shortening the bout durations making up 50% of total SB. With regards to L_F_, this morphology outcome appeared to become longer with ‘better’ daily SB patterns, which is a likely and positive result to note. L_F_ is a major determinant of maximum shortening velocity [15], with 50% of the differences in maximum shortening velocity between young and old adults explained by a reduction in GM L_F_ [64]. Moreover, improved gait speed following SB displacement (with LIPA) in older adults, is partially dependant on an increase in GM Lf [31]. This means that more breaks in sitting are suggested to have a positive impact on muscle architecture (specifically Lm, V_M_ and L_F_) and should therefore be recommended to the older population (for example in the health Apps available to them) rather than focussing on total sedentary time.

### Study strengths and limitations

Our sample exhibited similar characteristics to the general adult UK population in terms of anthropometrics [65] and gender distribution in older people [66]. In addition, SB/PA levels and adherence to current UK PA guidelines were in line with existing reports [67–70]. This confirms that our sample had similar characteristics to the general UK older adult population.

The GM muscle was chosen because it has been studied frequently regarding muscle architecture and size, and ultrasound scanning of it has been proven valid [71]. Moreover, GM is also an important muscle for postural balance in older adults [2] and hence physical functioning. It is an antigravity muscle, which shows fast impact of unloading (atrophy) as suggested in sedentary behaviour (SB). In addition, muscle volume quantification approaches using several MRI Scans yield results within the range of our study though direct comparison is difficult owing to inherent between-population differences (age, ratio of males:females, habitual physical activity) as well as differences in reporting (we report the median whilst other studies reported the mean) [72, 73].

The current study included all the spectrum of daily sedentarism including sleep. Indeed, levels of sleep and PA, decrease with ageing and might have opposed associations with musculoskeletal health [74]. In fact, sleep was previously identified as a risk factor for sarcopenia in older adults [75, 76]. Moreover, Piovezan et al. [25] have suggested that anabolic hormone cascades are inhibited, while catabolic pathways are enhanced in the skeletal muscle, due to age-related sleep problems. Given that sleep, SB and PA are partly co-dependent within a daily composition and (potentially) have independent effects on musculoskeletal health, it is a strength of our study to have taken all into account when studying the true associations between SB and muscle-tendon properties in the elderly.

Having good-to-excellent ICC, SE and TE values for most (4 out of 6) of the muscle outcomes tested in the current investigation showed that the collected data was reliable. Although, the remaining two outcomes (L_F_ and θ) showed lower ICCs of 0.700 and 0.645 respectively, these values could still be interpreted as moderate reliability. Therefore, the data in this investigation was generally regarded as being of acceptable quality, which is a major strength.

A notable limitation nonetheless was that ultrasound accuracy to assess muscle volume has not been validated in obese people [77]. We also did not quantify the intrinsic GM muscle composition in terms of any fatty or collagenous tissue infiltration. Furthermore, the current report is a cross-sectional design and as such may not infer any causal links between SB and muscle architecture, nor does it investigate any association between functional abilities and SB. Future studies should include longitudinal study designs and these outcomes in view of further enhancing our understanding of the impact of sedentary behaviour (independent of other concurrent physical behaviours) on musculoskeletal characteristics. Finally, as we did not check the normal distribution (homoscedasticity) of the residuals owing to the large number of models included in this study (n = 20), there could be a potential (which arguably is minimal given the number of observations per outcome variable [78, 79]) for the models to not be equally powered across the spectrum.

## Conclusion

The current investigation provides a novel identification of a number of associations between some aspects of resting GM size and architecture, and SB in older adults. Notably, it was not mostly accumulated daily SB but SB daily pattern (i.e. breaks and bouts duration) that was associated with the size, morphology and architecture of the GM. There are therefore, implications for physical behaviour guidelines on ensuring SB bouts are kept short, and interrupted with LIPA through the day, to offset the ageing-related loss of muscle mass. What the implication of relationships and exact associations with other GM outcomes will be, such as muscle force generating capacity, is yet to be determined.

## Supporting information

S1 Table Correlation coefficients during covariate analysis. L_MTU_, muscle-tendon unit length; L_M_, muscle length; ACSA, anatomical cross-sectional area; V_M_, muscle volume; L_F_, fascicle length; L_F-N_, normalised fascicle length; θ, fascicle pennation angle; PCSA, physiological cross-sectional area; BMC, bone mineral content; Skeletal muscle index; FRAT, falls risk assessment tool; RA, rheumatoid arthritis; ^¶^Log-transformed. Bold values represent significances at P<0.05 level.
(PDF)

S1 Data (XLSX)
