# Supplementary material for: Sedentary behaviour (especially accumulation pattern) has an independent negative impact on skeletal muscle size and architecture in community-dwelling older adults
Source: PLoS One. 2024 Feb 23;19(2):e0294555. doi: 10.1371/journal.pone.0294555 (PMC10889859; doi:10.1371/journal.pone.0294555)
Supplement: S1 Table — LMTU, muscle-tendon unit length; LM, muscle length; ACSA, anatomical cross-sectional area; VM, muscle volume; LF, fascicle length; LF-N, normalised fascicle length; θ, fascicle pennation angle; PCSA, physiological cross-sectional area; BMC, bone mineral content; Skeletal muscle index; FRAT, falls risk assessment tool; RA, rheumatoid arthritis; ¶Log-transformed. Bold values represent significances at P<0.05 level. (PDF) [file pone.0294555.s001.pdf]

Table S1: Correlation coefficients during covariate analysis. L_{MTU}, muscle-tendon unit length; L_M, muscle length; ACSA, anatomical cross-sectional area; V_M, muscle volume; L_F, fascicle length; L_{F-N}, normalised fascicle length; θ, fascicle pennation angle; PCSA, physiological cross-sectional area; BMC, bone mineral content; Skeletal muscle index; FRAT, falls risk assessment tool; RA, rheumatoid arthritis; ¶Log-transformed. Bold values represent significances at P<0.05 level.

	L_{MTU}	L_M	ACSA25¶	ACSA50¶	ACSA75¶	V_M¶	L_F	L_{F-N}	θ	PCSA¶
Ethnicity	-0.156	-0.115	-0.101	-0.078	-0.156	-0.140	-0.138	-0.046	-0.079	-0.085
SMI	**0.489**	**0.347**	**0.431**	**0.490**	**0.482**	**0.543**	**0.193**	-0.116	**0.295**	**0.555**
Fat mass	**-0.458**	**-0.263**	0.085	0.137	0.021	-0.036	-0.089	0.127	0.129	-0.003
Lean mass	**0.450**	**0.257**	-0.081	-0.137	-0.020	0.035	0.086	-0.125	-0.117	0.002
BMC mass	**0.397**	**0.252**	-0.097	-0.101	-0.022	0.042	0.090	-0.116	**-0.225**	0.012
Adiposity class	0.062	-0.054	**0.304**	**0.409**	**0.282**	**0.270**	0.026	0.037	**0.289**	**0.310**
FRAT score	-0.158	**-0.195**	-0.125	-0.148	-0.178	**-0.219**	-0.156	0.005	0.035	-0.167
Menopause age	0.050	0.128	0.039	-0.232	**-0.280**	-0.075	0.058	-0.080	-0.141	-0.121
Major illness history	**0.232**	0.159	0.025	0.143	0.152	0.161	-0.039	-0.170	-0.004	**0.208**
Statins usage	0.123	0.009	-0.005	0.002	0.073	0.021	0.039	0.029	0.017	0.002
Smoking	**-0.199**	**-0.241**	-0.158	-0.072	-0.022	-0.179	-0.116	0.124	0.002	-0.150
Resistance training	-0.005	0.124	0.083	-0.030	-0.048	0.051	**0.213**	0.104	-0.181	-0.053
Dairy products	-0.041	0.039	0.040	-0.071	-0.076	-0.021	**-0.244**	**-0.273**	0.143	0.104
Caffeine intake	0.150	0.090	0.063	-0.008	0.030	0.059	0.099	0.016	-0.171	0.018
RA diagnosis	0.086	0.079	0.082	**0.195**	**0.219**	0.178	0.005	-0.058	0.084	**0.204**
Daily alcohol intake ≥3 units	0.188	0.155	0.024	0.100	0.161	0.150	0.056	-0.085	0.010	0.168
Calcium/vitamin D supplements	**-0.205**	-0.080	-0.079	-0.101	-0.090	-0.110	-0.010	0.048	-0.052	-0.128
